# No effects of a 4-week post-exercise sauna bathing on targeted gut microbiota and intestinal barrier function, and hsCRP in healthy men: a pilot randomized controlled trial

**DOI:** 10.1186/s13102-022-00497-z

**Published:** 2022-06-16

**Authors:** Joanna Karolkiewicz, David C. Nieman, Tomasz Cisoń, Joanna Szurkowska, Mirosława Gałęcka, Dariusz Sitkowski, Zbigniew Szygula

**Affiliations:** 1 Department of Food and Nutrition, Poznan University of Physical Education, Poznań, Poland; 2 Department of Biology, Appalachian State University, North Carolina Research Campus, Kannapolis, NC USA; 3 Department of Physiotherapy, Institute of Physical Education, State University of Applied Sciences in Nowy Sącz, Nowy Sącz, Poland; 4 Institute of Microecology, Poznań, Poland; 5 Department of Physiology, Institute of Sport - National Research Institute PL, Warsaw, Poland; 6 Department of Sports Medicine and Human Nutrition, University of Physical Education, Kraków, Poland

**Keywords:** Gut microbiota, Exercise, Sauna bathing, Intestinal barrier function

## Abstract

**Background:**

Body temperature fluctuations induced by acute exercise bouts may influence the intestinal barrier with related effects on epithelial permeability, immune responses, and release of metabolites produced by the gut microbiota. This study evaluated the effects of post-exercise sauna bathing in young men undergoing endurance training on gut bacteria inflammation and intestinal barrier function.

**Methods:**

Fifteen (15) untrained males aged 22 ± 1.5 years were randomly assigned to exercise training (ET) with or without post-exercise sauna treatments (S). Participants in the group ET + S (n = 8) exercised 60 min, 3 times per week, on a bicycle ergometer followed by a 30-min dry Finish sauna treatment. The control group (ET, n = 7) engaged in the same exercise training program without the sauna treatments. Blood and stool samples were collected before and after the 4-week training program. Blood samples were analysed for the concentration of high-sensitivity C-reactive protein (hsCRP) and complete blood counts. Stool samples were analysed for pH, quantitative and qualitative measures of targeted bacteria, zonulin, and secretory immunoglobulin A.

**Results:**

Interaction effects revealed no differences in the pattern of change over time between groups for the abundance of selected gut microbiome bacteria and stool pH, zonulin, sIgA, and hsCRP. Pre- and post-study fecal concentrations of *Bifidobacterium spp., Faecalibacterium prausnitzii,* and *Akkermansia muciniphila* were below reference values for these bacteria in both groups.

**Conclusions:**

The combination of 4-weeks exercise followed by passive heat exposure did not have a measurable influence on targeted gut microbiota, intestinal barrier function, and hsCRP levels in young males.

***Trial registration*:**

The study was retrospectively registered in the clinical trials registry (Clinicaltrials.gov) under the trial registration number: NCT05277597. Release Date: March 11, 2022.

## Background

The processing of signals from the internal and external environment depends on the genetic capabilities of microorganisms colonising the human body. This process involves the switching on or off of specific genes and their replacement or silencing, which subsequently causes the microorganisms to acquire new properties that help them adapt to the altered environment. On the other hand, the immune system controls, regulates, and evaluates the microorganisms residing in the human body on a continuous basis [1]. These interactions take place in the border zone, at the interface of bacteria, intestinal epithelial cells, dendritic cells, lymphocytes, and granulocytes. This multidirectional network enables signal transmission and communication between the gut microbiota and the host, and has implications for how the immune system responds to athletic endeavor [2].

High exercise workloads may negatively influence the intestinal mucosa and barrier function, the gut microbiota composition, and blood flow leading to immune dysfunction in athletes [3]. During intense exercise, blood flow is directed away from the gastrointestinal tract to support skeletal muscle oxygen demand. Intestinal ischemia, measured through gastric tonometery, has been shown to occur within 10 min of high-intensity exercise [4]. This ischemic environment leads to tight junction (TJ) protein breakdown as measured with changes in occludin and claudins [5].

A rise in core temperature and dehydration during long-duration exercise may also influence the intestinal barrier with related effects on epithelial permeability and release of metabolites produced by the gut microbiota [6]. High temperature imposes physiological adaptations including reduced blood flow to the gastrointestinal tract, which triggers a hypoxic environment and protein breakdown that leads to intestinal epithelial cell damage. On the basis of cell culture studies, heat stress causes an increase in TJ permeability that is associated with some changes in TJ protein expression including an increase in occludin and a decrease in zonula occludens-1 (ZO-1) [7]. Heavy exercise, especially marathon running, is known to cause gastrointestinal blood loss, gastritis, and colitis, which are in part attributed to gastrointestinal ischemia [8]. Induced mucosal inflammation causes translocation of smaller molecules from the leaky gut, which activates multiple signaling pathways that stimulate inflammatory immune responses. Findings suggest that increased sauna dehydration and body temperature may also be sufficient to induce intestinal permeability [9].

Environmental temperature and heat stress have a major impact on the homeostatic control of the microbiome. Shifts in core body temperature can impact microbiome function because of the sensitivity of the gut epithelium to temperature, and this may lead to dysbiosis in some athletes [10]. A variety of sauna bathing therapies are being applied in sport for exercise recovery, treatment of inflammation, improvement in cardiovascular stability, fluid balance, and thermal tolerance [11, 12]. The impact of post-exercise sauna on the gut microbiome remains unknown.

Evidence suggests that intensive exercise performed at high temperature can alter the gut microbiota and intestinal epithelial barrier function [5]. The underlying mechanisms by which heat stress may alter intestinal permeability are not fully understood. Exercise-induced elevations in body temperature shunts blood from the gastrointestinal tract to the skin compartment to facilitate heat loss [13]. Blood flow is also redirected to the working muscles resulting in reduced total splanchnic perfusion and gastrointestinal ischaemia. This may exert tensional stress on tight junctions leading to disturbances in intestinal barrier and transport protein function, secretory IgA (sIgA) production, and levels of mucins, antimicrobial molecules, and cytokines involved in zonulin regulation [4, 14].

Taken together, these data imply that sauna bathing applied immediately after a physical training session may impact homeostatic control of the gut microbiota and the function of the intestinal barrier. This study evaluated the effects of post-exercise sauna bathing in young men undergoing endurance training. We hypothesized that the combination of cycling in mild heat stress with post-exercise sauna treatments would result in greater disturbances in gut microbiota, gut permeability, and inflammation compared with cycling in mild heat stress.

## Methods

### Participants

Study participants included 15 young healthy, Caucasian males aged 22.0 ± 1.5 years, physical education and physiotherapy students. All patients underwent routine blood tests, ECG and medical examinations. Also, eligibility tests to detect possible contraindications to repeated sauna treatments were carried out by a physician who was a member of the research team. The study inclusion criteria included voluntary written consent to participation in a 4-week physical training and a series of sauna treatments, absence of medical contraindications such as epilepsy, addiction to medicines, alcohol and drugs, cancer, blood clotting disorders, no infections, and no injuries in the last 4 weeks prior to the study. Exclusion criteria including the intake of antibiotics, steroids, oral antifungal agents (except for topical antifungals), antiparasitic agents, pre- and/or probiotics, history of travel to tropical countries during the last 4 weeks before the study, and history of adverse responses to sauna bathing.

Participant did not use the sauna regularly and were not in the sauna 4 weeks earlier.

During intervention periods the participants maintained their normal physical activity as provided by their academic program as well as their own activity for recreational purposes.

The study participants were informed about the aim and methods used in the study. They were also informed about the possibility to withdraw from the study at any stage of the project and they had insight into their results. All participants consented to participate in the study in accordance with the Declaration of Helsinki. The project was approved by the Ethics Committee for Human Research at the Poznań University of Medical Sciences (*approval no.* *173/16 of 4 February 2018*).

### Procedure

This study utilized a randomized, parallel group design. The participants were randomly assigned to exercise training (ET) without or with post-exercise sauna treatments (ET + S) by using a permuted blocks of block size 4. design with a computer. Participants in the group ET + S (n = 8) exercised 60 min, 3 times per week, on a bicycle ergometer followed by a 30-min dry sauna treatment. The control group (ET, n = 7) engaged in the same exercise training program without the sauna treatments. The 60-min exercise bouts were performed on calibrated Keiser M3 ergometers (Germany). The initial exercise intensity was set at 50% VO_2_peak for 2 weeks, and then increased to 60% for the final two weeks of training. The physical exercise was performed in controlled mild heat stress (temperature of 22–23 °C, and relative humidity of 30–33%). Immediately after finishing the 60-min exercise bout, subjects from group ET + S spent 30 min in a dry sauna (in the sitting position), at a temperature of approximately 90 °C at the chest level and relative humidity of air 10 ± 2%. The sauna treatment was divided into three or two parts (e.g. 3 × 10 min—for habituation during first two weeks, and 2 × 15 min during the next two weeks), and subjects were allowed to cool the body for a maximum of 3-min (e.g. by taking a cold shower and immersing the body in cool water up to the armpits). The use of fans, the consumption of cold drinks, and other methods of cooling during exercise were not allowed during the exercise without (ET) and with sauna (ET + S) since the aim was to provoke dehydration and a corresponding decrease in blood volume and splanchnic perfusion with gut hypoxia.

Rectal temperature of the subjects was measured with ELLAB DM852 (Dania) Electrothermometer during the 1st and the last exercise training in group ET and during exercise and sauna bathing in group ET + S. The mean rectal temperature (Tre) in the ET group 37.1 °C ± 0.2 before training and 38.3 °C ± 0.3 after training, while in the ET + S group was 36.9 °C ± 0.1 before training and 38.9 °C ± 0.4 after training and sauna. Mean rectal temperature increased by 1.2 °C in group ET and by 2 °C in group ET + S.

Study participants were told to maintain their normal dietary patterns during the study, and this was verified with 3-day food records (2 week days, 1 weekend day) at the beginning and end of the 4-weeks study. Energy and macronutrient intake was assessed using the NUVERO dietary assessment system (Poland). Daily intake of energy, protein, carbohydrates, fats and fibre during the study were constant and comparable between the groups. The participants were also instructed to maintain their normal physical activity and not to use any dietary supplements.

### Peak oxygen uptake

Peak oxygen uptake (VO2peak) was assessed with MetaMax 3B analyzer (Cortex, Germany) before and after the 4-week study using a graded exercise test (GXT) with a cycloergometer Cyclus2 (Avantronic, Germany). Metabolic measurements included ventilation (VE), oxygen consumption (VO2), carbon dioxide production (VCO2), and heart rate (HR). GXT was conducted on a Cyclus2 cycle ergometer (RBM elektronik-automation GmbH, Germany). The test started with a 10-min warm-up period: 5-min at a workload of 75 W and next 5 min at 130 W. Immediately after the warm-up, the workload was increased by 25 W every minute until voluntary exhaustion or when the participant was unable to maintain a pedaling frequency over 50 rpm. At the final stage of the test, the participants were verbally encouraged by the investigators to achieve maximal effort. Breath-by-breath values of ventilation, oxygen consumption, carbon dioxide production (averaged then over 30 s) were measured by a MetaMax 3B gas analyzer (Cortex, Biophysik GmbH, Germany). The gas sensors and the flowmeter were calibrated before each test. The two highest consecutive 30-s values of VO_2_ were averaged to determination of VO_2peak_ [15].

### Body composition assessment

Body mass, height, and body composition were measured before and after the 4-week study. The subjects were weighed naked using a certified medical digital beam scale WB-3000 (TANITA Corporation, Tokyo, Japan) before and after exercise training in group ET and before and after exercise training and after sauna in group ET + S. Body composition was measured in the fasting state using a GE Lunar Prodigy Primo Full Densitometer with enCore Body Composition option (GE Healthcare Technologies, USA).

### Blood and stool sample collection

Blood and stool samples were collected before and after the 4-week study. Blood samples (approx. 2 ml) were taken from the antecubital vein and centrifuged at 4000 rpm and 4 °C. The serum was separated from the sample and stored at − 70 °C. The concentration of high-sensitivity C-reactive protein (hsCRP) was measured by immunoenzymatic assay using a commercially available kit (DRG International Inc., Springfield Township, NJ, USA; test sensitivity: 0.1 mg/L and 5 ng/mL). Complete blood count indices were determined by flow cytometry with a Synergy 2 SIAFRT analyser (Bio Tek, Winooski, VT, USA).

In order to perform qualitative analyses of selected indicator bacteria in the gastrointestinal tract, and to determine the stool pH, the studied men were requested to provide a stool sample within 24 h of collection.

Stool samples for analysis were collected twice at the beginning and end of the training period, and stored at refrigerator temperature (2–8 °C). Stool sample collection was performed according to the established protocol developed by KyberKompaktPRO (Institute of Microecology). To this end, a 150-ml sterile container was to be filled to three quarters of its volume with material preferably taken from eight different locations, and closed tightly with a lid. The indicator bacteria, sIgA (marker of mucosal immunity), and the concentrations of zonulin (marker of intestinal permeability) in stool were evaluated before and after completing the training programme in both studied groups of men.

Bacterial DNA was isolated from stool samples using the QIAamp Fast DNA Stool Mini Kit (QIAGEN, Danish). An appropriate quantity of stool was weighed into a sterile tube. The isolation of bacterial DNA from the stool sample was performed according to the manufacturer's protocol. The DNA eluates were stored frozen until subsequent analyses.

The anaerobic bacteria including *Faecalibacterium prausnitzi* of the genus *Faecalibacterium, Akkermansia muciniphila* of the genus *Akkermansia, Bifidobacterium spp.* of the genus *Actinobacteria*, and *Bacteroides spp*. of the genus *Bacteroidetes* were determined by Real-Time PCR with appropriate primers (ThermoFisher Scientific, USA) (Table 1). The reaction mixture contained QuantiFast SYBR Green PCR Kit (Qiagen), RNase-free water (Qiagen), and a mixture of forward and reverse primers selected for the bacteria tested. The analyses were conducted in an ABI 7300 analyser (ThermoFisher Scientific, USA).

**Table 1:** Specific primers used for the determination of different microorganisms

Name	Product description	Sequence
Praus-F480	*Faecalibacterium prausnitzii* forward starter	CAGCAGCCGCGGTAAA
Praus-R631	*Faecalibacterium prausnitzii* reverse starter	CTACCTCTGCACTACTCAAGAAA
Akk.muc-F	*Akkermansia muciniphila* starter forward	CAGCACGTGAAGGTGGGGAC
Akk.muc-R	*Akkermansia muciniphila* starter reverse	CCTTGCGGTTGGCTTCAGAT
F-Bifid09c	*Bifidobacterium spp.* forward starter	CGGGTGAGTAATGCGTGACC
R-Bifid06	*Bifidobacterium spp*. reverse starter	TGATAGGACGCGACCCCA
Bacter11	*Bacteroides spp.* forward starter	CCTWCGATGGATAGGGGTT
Bacter08	*Bacteroides spp.* starter reverse	CACGCTACTTGGCTGGTTCAG
Uni-F340	Starter universal forward	ACTCCTACGGGAGGCAGCAGT
Uni-R514	Starter universal revers	ATTACCGCGGCTGCTGGC

The final bacterial count/g of stool was obtained by converting the number of copies of the sequence amplified by PCR in the bacterial genome (for *Faecalibacterium, Akkermansia muciniphila, Bifidobacterium spp.*, and *Bacteroides spp.*, respectively) and the dilution factor applicable to the kit used for DNA isolation from stool samples.Table 2 presents the standards used in the studies.

**Table 2:** Standards applied for the determination of different microorganisms

Name	Among of DNA (copies/ml)	Product description
*Bifidobacterium infantis* DNA	5e8	Standard in identification of *Bifidobacterium* spp., isolated from *Bifidobacterium infantis*
*Bacteroides fragilis* DNA	2e9	Standard in identification of *Bacteroides* spp., isolated from *Bacteroides fragilis*
*Faecalibacterium prausnitzii* DNA	7,8e8	Standard in identification of *Faecalibacterium prausnitzii*
*Akkermansia muciniphila* DNA	3,9e8	Standard in identification of *Akkermansia muciniphila*

The limit of detection for the evaluated parameters was 10^2^ [CFU/g of feces]. For values below 10^2^ [CFU/g of feces] [cut-off point], the value of 0 was adopted for statistical analysis, which, however, does not mean that the test sample was bacteria-free. The results of quantitative bacterial analysis were converted to the decimal logarithm (Log10). The entire Real-Time PCR methodology was developed and validated by the *Institute of Microecology* in Herborn, Germany. Reference values for selected indicator bacteria and stool pH are presented in Table 3.

**Table 3:** Reference values for selected indicator bacteria and stool pH

Species [Genus]	Standard [Log10 CFU/g feces]	Method
ANAEROBIC		
*Bifidobacterium spp.*	≥ 8	Real-time PCR
*Bacteroides spp.*	≥ 9	Real-time PCR
*Faecalibacterium prausnitzii*	≥ 9	Real-time PCR
*Akkermansia muciniphila*	≥ 8	Real-time PCR
Feces pH	5.8–6.5	

The concentrations of sIgA (marker of mucosal immunity), and zonulin (marker of intestinal permeability) in stool were evaluated before and after completing the training programme in both groups. The evaluation of stool zonulin and secretory immunoglobulin A (sIgA) concentrations required sample extraction. A stool extract was prepared using stool collection devices (Stool Sample Application System – SAS, K6998SAS) filled with 0.75 ml of washing buffer warmed to room temperature. Each stool sample was vortexed for homogeneity. In the next step, a stool collection device was inserted into the sample, so that all grooves in the device were filled with stool (15 mg), vortexed and analysed. Zonulin concentrations were assessed using the IKD Zonulin ELISA Kit (Immunodiagnostik AG, Bensheim, Germany). The minimum sensitivity of the test was 0.241 ng/mL; borderline value > 78,0 ng/ml stool.

Secretory immunoglobulin A concentrations in stool samples were determined with the Secretory IgA test (ImmuChrom GmbH, Heppenheim, Germany). The minimum sensitivity of the test was 3.1 ng/ml.

The concentrations were measured by means of an immunoenzymatic method (ELISA) with a BioTek PowerWave XS spectrophotometer (USA).

### Statistical analysis

Data were presented as means and standard deviations (SD). The Shapiro–Wilk test was used to check the data for normality of distribution. Assumption on sphericity was tested using Mauchley’s test, verifying if variances of certain variables were identical and equal to respective co-variances. A two-way analysis of variance (ANOVA) for repeated measures was used to analyze the differences in the effect of time, group and time x group. Bonferroni post hoc tests were performed to assess the significance of differences between pairs of measurements. A *p*-value < 0.05 was considered significant. For statistically significant results, the coefficients η^2^ are presented as an indicator of the effect size. Statistical analyses were performed using the Statistica 13.3 software package (TIBCO Software Inc., Palo Alto, CA, USA (2017) version 10.

## Results

An overview of baseline characteristics and VO_2peak_ in the ET and ET + S group healthy men are given in Table 4. Participants` demographics and VO_2peak_ were comparable between the two groups. The changes in body mass, percentage of body fat and VO_2peak_ did not differ between the groups after the 4-week study (Table 4).

**Table 4:** Pre- and post-study body mass, body fat and VO_2peak_ in the ET group and the ET + S group

	Pre-study		Post-study		2-way ANOVA (*p*-value;η^2^)		
	ET Mean ± SD	ET + S Mean ± SD	ET + S Mean ± SD	ET Mean ± SD	Group	Time	Group x Time
Body mass (kg)	79.0 ± 5.2	77.5 ± 9.9	79.9 ± 5.9	78.0 ± 9.0	0.685	0.079	0.553
Body fat (%)	19 ± 5.3	21.3 ± 3.5	18.6 ± 4.7	20.3 ± 3.7	0.371	**0.028** **(0.32)**	0.361
VO_2 peak_ (ml/kg/min)	51.6 ± 5.5	48.2 ± 8.2	50.8 ± 6.3	49.9 ± 8.8	0.584	0.215	0.150

*p* < 0.05 were in bold

η^2^ Effect size, VO_2peak_ Peak oxygen uptake

Body mass dropped by 0.55 ± 0.13 kg in ET after the 1st training session, 0.85 ± 0.24 kg after the 6th training session, and 1.05 ± 0.22 kg after the last training session. Body mass changes in ET + S were greater and dropped by 1.89 ± 0.4 after the 1st training session, 2.07 ± 0.45 after the 6^th^ training session, and 1.86 ± 0.25 kg after the last training session.

This study did not include a non-exercise control group and therefore was not designed to investigate exercise training effects on targeted gut microbiota, intestinal permeability, or inflammation. Instead the primary focus was on the influence of post-exercise sauna bathing on gut microbiota during a 4-week exercise training period.

Interaction effects revealed no differences in the pattern of change over time between groups for the abundance of selected gut microbiome bacteria and stool pH (Table 5).

**Table 5:** Pre- and post-study targeted gut microbiota and stool pH in the ET group and the ET + S group

Gut bacteria [Log_10_ CFU/g feces]	Pre-study		Post-study		2-way ANOVA (*p*-value)		
	ET Mean ± SD	ET + S Mean ± SD	ET Mean ± SD	ET + S Mean ± SD	Group	Time	Group x Time
*Bifidocacterium* spp.	6 ± 0.8	6.2 ± 0.8	6.4 ± 0.9	6.2 ± 0.8	0.924	0.399	0.562
*Bacteroides* spp.	8.9 ± 0.3	8.6 ± 0.5	9 ± 0.6	8.7 ± 0.7	0.201	0.502	0.871
*F. prausnitzii*	8.5 ± 0.3	8.5 ± 0.5	8.5 ± 0.4	8.4 ± 0.7	0.885	0.493	0.531
*A. muciniphila*	3.4 ± 1	4.1 ± 2.3	4 ± 1.9	4.2 ± 2.2	0.668	0.084	0.210
Stool pH	6.1 ± 0.5	6.3 ± 0.3	6.4 ± 0.4	6.3 ± 0.7	0.982	0.524	0.326

During a 4-week exercise training period, significant time-effects on stool zonulin (*p* < 0.05) were noted. Interaction effects revealed no differences in the pattern of change over time between groups for stool zonulin, sIgA, and hsCRP (Table 6).

**Table 6:** Pre- and post-study zonulin, sIgA, and hsCRP in the ET group and the ET + S group

	Pre-study		Post-study		2-way ANOVA (*p*-value; η^2^)		
	ET Mean ± SD	ET + S Mean ± SD	ET Mean ± SD	ET + S Mean ± SD	Group	Time	Group x Time
Zonulin (ng/ml)	132 ± 119	119 ± 80	289 ± 183	228 ± 179	0.577	**0.005** **(0.46)**	0.565
sIgA (µg/ml)	1653 ± 1390	1385 ± 1408	1059 ± 889	1784 ± 1213	0.698	0.745	0.114
hsCRP (ng/dl)	0.7 ± 1.2	1 ± 1	1.4 ± 2.5	0.4 ± 0.5	0.603	0.785	0.054

*p* < 0.05 were in bold

η^2^ Effect size, sIgA Secretory immunoglobulin A, hsCRP High-sensitivity C-reactive protein

In the ET + S group, there was a significantly higher level of monocytes before the beginning of the training period compared to the ET group (*p* < 0.05). During the 4-week exercise training period, significant time-effects on subsets of neutrophils (*p* < 0.01) and lymphocytes level (*p* < 0.05) were noted. In the ET + S group there was an increase of lymphocytes level after a 4-week exercise training, while in the ET group a reduction in lymphocytes level was observed after training when compared to the pre-study. (Table 7).

**Table 7:** Pre- and post-study white blood cell counts (WBC) and subsets the ET group and the ET + S group

Blood cell count [10^9^/L]	Pre-study		Post-study		2-way ANOVA (*p*-value; η^2^)		
	ET Mean ± SD	ET + S Mean ± SD	ET Mean ± SD	ET + S Mean ± SD	Group	Time	Group x Time
White blood cells	6.6 ± 1	7.9 ± 3.6	5.8 ± 1.2	6.5 ± 1.5	0.322	0.054	0.606
Neutrophils	3.3 ± 0.5	4.7 ± 3.5	2.6 ± 0.9	3.2 ± 1	0.405	**0.009 (0.46)**	0.923
Lymphocytes	2.4 ± 0.8	2.1 ± 0.3	2.3 ± 0.7	2.3 ± 0.4	0.140	**0.033 (0.3)**	0.848
Monocytes	0.5 ± 0.1	0.8 ± 0.2	0.5 ± 0.2	0.7 ± 23.4	**0.044 (0.6)**	0.591	0.145
Eosinophils	0.2 ± 0.1	0.2 ± 0.2	0.2 ± 0.2	0.3 ± 0.4	0.887	0.070	0.604
Basophils	0.04 ± 0.1	0.03 ± 0.01	0.04 ± 0.01	0.04 ± 0.0	0.064	0.001	0.178

*p* < 0.05 were in bold

η^2^ Effect size, WBC Total white blood cells

## Discussion

In this study, participants exercised 60 min for three times per week over a 4-week period with or without 30-min post-exercise dry sauna treatments. Blood and stool samples were collected pre- and post-study. The key finding of the present study is that repeated applications of sauna bathing just after physical training had no effect on targeted gut microbiota, intestinal permeability marker—zonulin, intestinal barrier function marker—sIgA, and inflammation marker- hsCRP in young adult males.

Environmental heat and exercise-induced elevations in temperature may influence the gut microbiome [6]. Given the potential for temperature shifts to modulate gut microbe function and composition, we hypothesized that the combination of exercise and sauna bathing would influence the intestinal barrier and targeted microbiota. Little is known as to which variable (heat or exercise intensity) plays a greater role in causing intestinal barrier dysfunction. Some studies reported increases in exercise-induced intestinal permeability with an average core temperature ranging from 38.2 to 39.6 °C [4, 16–18]. Intensive and prolonged exercise can also induce intestinal hypoperfusion, dehydration, impaired osmolarity of body fluids and gut motility, and increased permeability of the intestinal barrier [19–21].

A healthy gut microbiota profile has many specific functions in host nutrient metabolism, maintenance of the structural integrity of the intestinal mucosal barrier,

immunomodulation and protection against pathogens. A recent review of the literature on the influence of exercise on the gut microbiota of healthy adults showed that short-term and medium/long-term exercise interventions influence the fecal counts of some phyla. However, the heterogeneity between studies hampered any strong conclusions from being drawn [22]. Recent findings by Bycura et al. [23] suggested that the human gut microbiome can change in response to cardiorespiratory but not resistance exercise training. In the present study, the abundance of the anaerobic bacteria including *Faecalibacterium prausnitzi* of the genus Faecalibacterium, *Akkermansia muciniphila* of the genus Akkermansia, *Bifidobacterium spp.* of the genus Actinobacteria, and *Bacteroides spp*. of the genus Bacteroidetes were measured. The findings of the present study did not support our hypothesis that the combination of sauna bathing and exercise with mild heat stress compared to exercise with mild heat stress alone had an influence on the abundance of labelled gut bacteria. Animal studies support that remodeling of the gut microbiome is responsive to chronic alterations in both ambient and internal temperature [24, 25]. For example, 24-week-old female mice exposed to 34 °C for 8 weeks experienced a significant alteration in microbial composition, with increases in several genera including Akkermansia [25]. However, human data, especially within the context of repeated acute thermal treatments are lacking [26].

Pre- and post-study fecal concentrations of *Bifidobacterium spp., Faecalibacterium prausnitzii, and Akkermansia muciniphila* were below reference values for these bacteria in both groups (Tables 3 and 5). Current evidence suggests that *Akkermansia muciniphila* and *Faecalibacterium prausnitzii* are highly abundant human gut microbes in healthy individuals, and reduced levels are associated with inflammation and alterations intestinal integrity [27]. Fecal abundance of *A. muciniphila* and *F. prausnitzii* may increase in response to physical training [28, 29], but our data did not confirm these findings. Others have reported lower levels of *F. prausnitzii* and *A. municiniphia* in the human intestine over the past few years [30].

The pH along the human colon usually varies from 5 to 7, and depends on fermentation processes, secretion of bicarbonate by colonic epithelial cells and absorption of microbial metabolites by host epithelial cells [31]. The pH levels determined in stool samples obtained from both studied groups of men, before and after the completion of training programmes were within the normal range (Table 5). Normal stool pH supports the maintenance of healthy and stable gut microbiota producing short-chain fatty acids, and may have protective functions against pathogenic bacteria, especially in healthy individuals [32].

Fecal zonulin is a recognized biomarker of intestinal permeability [20]. An important factor which activates the production of zonulin is intestinal ischaemia developing during intense physical exercise. Intensive exercise with environmental heat stress may contribute to damage to the gut mucosa and the intestinal barrier. Moreover, prolonged exercise increases stress hormone levels and LPS translocation, resulting in increased pro-inflammatory cytokine levels and intestinal permeability [33]. Stool zonulin concentrations decreased in both groups during the 4-week exercise training period indicating no added effect of post-exercise sauna bathing (Table 6). Stool zonulin levels were somewhat elevated in both groups of men before the initiation of training programmes reflecting other influences including high intake of gluten-containing foods, bacterial infections, or high levels of mental stress [34].

Secretory IgA plays an important role in mucosal immunity, can survive in harsh environments such as the GI, and provides first-line protection against potentially pathogenic microbes [35]. Gut sIgA imbalances have been lined to various diseases [24]. Lifestyle, exercise, stress, and diet can influence sIgA levels [25]. We observed faecal sIgA levels within the reference range and changes during the 4-week study did not differ between ET + S and ET groups (Table 6). Several gut microbiota species may be elevated in athletes, including *Enterococcus spp.*, *Lactobacillus spp. and Bacteroides spp.* These may have an impact on intestinal inflammation by increasing the expression of tight junction proteins, but may also increase the production and secretion of mucin and antimicrobial peptides (AMPs) that combat pathogenic invasion and stimulate plasmocytes for IgA secretion [36]. Our data indicated that 4-weeks of exercise training with or without sauna bathing had no influence on sIgA secretion by GI epithelial cells or changes in leukocyte subsets and hsCRP (Tables 6–7).

## Limitation of the study

This study had several limitations. A randomized parallel group design was employed, and subject numbers were low for this type of investigation. In addition, only men were enrolled in the study. The range of inflammatory biomarkers measured is limited to hsCRP levels. The exercise training program and sauna treatments included just 12 sessions during a 4-week program, and this may not have been a sufficient physiological stimulus to induce change in the measured outcomes, especially in young adult males.

## Conclusions

The combination of 4-weeks exercise followed by passive heat exposure did not have a measurable influence on targeted gut microbiota, intestinal barrier function, and hsCRP levels in young males.

## Data Availability

The datasets generated during and/or analysed during the current study are available from the corresponding author on reasonable request.

## Abbreviations

AMPs: Antimicrobial peptides
GI: Gastrointestinal
hsCRP: High-sensitivity C-reactive protein
LPS: Lipopolysaccharide
η^2^: effect size
sIgA: Secretory immunoglobulin A
TJ: Tight junction
WBC: Total blood white blood cell
VO2peak: Peak oxygen uptake
